# Factors Related to Non-compliance With Non-pharmaceutical Interventions to Mitigate the Spread of SARS-CoV-2: Results From a Survey in the Swiss General Adult Population

**DOI:** 10.3389/fpubh.2022.828584

**Published:** 2022-03-25

**Authors:** Michael P. Hengartner, Gregor Waller, Agnes von Wyl

**Affiliations:** School of Applied Psychology, Zurich University of Applied Sciences, Zurich, Switzerland

**Keywords:** SARS-CoV-2, COVID-19, non-pharmaceutical interventions, compliance, public health measure

## Abstract

**Background:**

Non-pharmaceutical interventions (NPI) play an important role in national efforts to control and contain the spread of SARS-CoV-2, but some people do not comply with these public health measures. The aim of this study was thus to describe this group of noncompliant people.

**Methods:**

A random sample of 1,157 people was drawn from the adult general population of Switzerland based on a three-stepped quota scheme considering the variables age (18–31, 32–45, 46–59, and ≥60 years), sex (male and female), and language region (German-, French-, and Italian-speaking Switzerland). We assessed a global scale of non-compliance with NPI based on several individual measures such as wearing face masks and social distancing. As predictor variables we included objective sociodemographic variables (e.g., age, sex) and easy measurable constructs (e.g., fears and worries about COVID-19, trust in medical experts).

**Results:**

Out of 14 predictor variables tested, seven were statistically significantly associated with increased non-compliance with NPI: male sex, younger age, self-identification as low-risk group, judging the consequences of an infection with SARS-CoV-2 as non-serious, less worries and fears about the pandemic, not obtaining regular information from health authorities, and not trusting in medical experts. The most parsimonious multivariable prediction model included the variables younger age, low appraisal of negative consequences, less fear and worries, not obtaining regular information from health authorities, and not trusting in medical experts. The model accounted for 27.9% of variance explained in non-compliance with NPI.

**Conclusion:**

Young adults who perceive COVID-19 as mostly harmless/inconsequential and who ignore and/or mistrust information from health authorities and medical experts, are the population most likely to be noncompliant with NPI. These findings may help to target a group of people at high risk of infection and to efficiently concentrate educational and interventional public health measures.

## Introduction

Non-pharmaceutical interventions (NPI) such as social distancing, wearing face masks, canceling of public events, and restrictions on private gatherings have been shown to be effective and play an important role in national efforts to mitigating the spread of SARS-CoV-2 infections (1–4). Although safe and effective vaccines are available (5–7), in various countries vaccination rates are rather low, especially in younger adults (https://graphics.reuters.com/world-coronavirus-tracker-and-maps/vaccination-rollout-and-access/). It has further been shown that vaccines are less effective in preventing infections with the predominant Delta variant and that protection against asymptomatic infections wanes quite rapidly after a few months, even though the vaccines still effectively prevent hospitalizations for severe COVID-19 (6, 8, 9). NPI thus complement national vaccination strategies, but not all people comply with them.

Previous research has consistently shown that younger age, male sex, low educational attainment, lack of trust in medical experts and science, and a underestimation of the harms/seriousness of COVID-19 are significantly associated with non-compliance with NPI (10–13). Nivette et al. previously examined non-compliance with NPI in a Swiss sample, but this study was restricted to people aged 22 years living in the city of Zurich (14). To the best of our knowledge, a comprehensive analysis of non-compliance with NPI in the Swiss general adult population has not been published thus far.

A reliable description of factors associated with non-compliance with NPI in the general Swiss adult population may help the Swiss government and public health authorities to effectively target prevention and awareness campaigns. The aim of the present study was thus to examine which individual factors are associated with non-compliance to NPI in the general adult population in Switzerland to better define this group at high risk of infection.

## Methods

### Sample Recruitment

A survey was conducted in collaboration with the market research institute Respondi. The Swiss online panel of Respondi comprises about 20,000 people broadly representative of the Swiss general population. Only people aged 18 and older were contacted to participate in the present survey. In total 2,515 people responded to the invitation by Respondi to participate. Sample recruitment was based on a three-stepped quota scheme considering the variables age (18–31, 32–45, 46–59, and ≥60 years), sex (male and female), and language region (German-, French-, and Italian-speaking Switzerland). Altogether 1,006 people were excluded because the quota size was already reached, and 352 people were excluded because they did not complete the questionnaire. Therefore, the final sample comprised 1,157 people representative of the Swiss adult population according to the distribution of age, sex, and region. All surveys were completed between December 11, 2020 and January 5, 2021. Formal approval by a national Ethics Committee was not required according to Swiss law as no health-related data were assessed.

### Measures

The survey assessed several constructs from the fields of media psychology, health psychology, personality psychology, and ecological psychology. The dependent variable was a global scale of non-compliance with NPI. This included the following public health measures: (1) If possible, I keep the necessary social distance to other people (1.5 m) in public; (2) When meeting friends or relatives, I keep the necessary social distance (1.5 m); (3) I wash my hands regularly; (4) If possible, I avoid public transportation; (5) If I have (cold) symptoms, I stay at home; (6) If I have (cold) symptoms, I make a SARS-CoV-2 test; (7) If possible, I avoid congregations of people; (8) If possible, I refrain from travels abroad; (9) I wear a mask in public when social distancing is not possible; (10) When I mix with people, I activate the Swiss COVID-19 tracing app; (11) I try to reduce private gatherings to a minimum. All items were rated on a five-point Likert scale ranging from 1 (“not at all true”) to 5 (“definitely true”). The global measure of non-compliance was built by computing the inverse mean score across all individual measures. Thus, the scale had a possible range from 1 (complete compliance with NPI) to 5 (complete non-compliance with NPI). The internal consistency of this scale was good (Cronbach's α = 0.82), but item 10 (activating the Swiss COVID-19 tracing app) was poorly correlated with the total scale score (corrected item-scale correlation: *r* = 0.28). Moreover, compliance with this measure was also very poor (50% indicated they would rather or definitely not activate the tracing app). After removing this item, the internal consistency of the scale was slightly improved (Cronbach's α = 0.84) and all items were moderately to highly correlated with the total scale score (range of corrected item-scale correlation: *r* = 0.38 to *r* = 0.68). For a list of all public health measures included (see Table 1).

**Table 1:** Non-compliance with non-pharmaceutical interventions to mitigate the spread of SARS-CoV-2 (*n* = 1157).

**Indicator**	**Response category**	***N*** **(%)**
If possible, I keep the necessary social distance to other people (1.5 m) in public	Definitely not true	19 (1.6%)
	Rather not true	33 (2.9%)
	Undecidedly true	41 (3.5%)
	Rather true	306 (26.4%)
	Definitely true	756 (65.3%)
	Missing	2 (0.2%)
When meeting friends or relatives, I keep the necessary social distance (1.5 m)	Definitely not true	67 (5.8%)
	Rather not true	145 (12.5%)
	Undecidedly true	125 (10.8%)
	Rather true	371 (32.1%)
	Definitely true	448 (38.7%)
	Missing	1 (0.1%)
I wash my hands regularly	Definitely not true	8 (0.7%)
	Rather not true	36 (3.1%)
	Undecidedly true	51 (4.4%)
	Rather true	273 (23.6%)
	Definitely true	784 (67.8%)
	Missing	5 (0.4%)
If possible, I avoid public transportation	Definitely not true	128 (11.1%)
	Rather not true	135 (11.7%)
	Undecidedly true	81 (7.0%)
	Rather true	248 (21.4%)
	Definitely true	559 (48.3%)
	Missing	6 (0.5%)
If I have (cold) symptoms, I stay at home	Definitely not true	31 (2.7%)
	Rather not true	88 (7.6%)
	Undecidedly true	106 (9.2%)
	Rather true	335 (29.0%)
	Definitely true	590 (51.0%)
	Missing	7 (0.6%)
If I have (cold) symptoms, a make a SARS-CoV-2 test	Definitely not true	200 (17.3%)
	Rather not true	146 (12.7%)
	Undecidedly true	236 (20.4%)
	Rather true	239 (20.7%)
	Definitely true	332 (28.7%)
	Missing	3 (0.3%)
If possible, I avoid congregations of people	Definitely not true	29 (2.5%)
	Rather not true	38 (3.3%)
	Undecidedly true	67 (5.8%)
	Rather true	264 (22.8%)
	Definitely true	755 (65.3%)
	Missing	4 (0.3%)
If possible, I refrain from travels abroad	Definitely not true	42 (3.6%)
	Rather not true	36 (3.1%)
	Undecidedly true	81 (7.0%)
	Rather true	173 (15.0%)
	Definitely true	822 (71.0%)
	Missing	3 (0.3%)
I wear a mask in public when social distancing is not possible	Definitely not true	34 (2.9%)
	Rather not true	41 (3.5%)
	Undecidedly true	37 (3.2%)
	Rather true	177 (15.3%)
	Definitely true	867 (74.9%)
	Missing	1 (0.1%)
I try to reduce private gatherings to a minimum	Definitely not true	56 (4.8%)
	Rather not true	100 (8.6%)
	Undecidedly true	102 (8.8%)
	Rather true	364 (31.5%)
	Definitely true	531 (45.9%)
	Missing	4 (0.3%)

As predictor variables we included only variables that can be assessed objectively (e.g., age, sex, educational attainment) or that are easily measurable with a few simple questions (e.g., fear and worries about COVID-19 pandemic, obtaining information from health authorities, trust in medical experts). The following variables were selected according to these criteria: (1) sex (male vs. female); (2) age (continuous measure in years); (3) nationality (Swiss vs. other); (4) educational attainment (low, medium, high corresponding broadly to high school, college, and higher education); (5) self-perceived high-risk group (yes vs. no based on age and chronic health conditions); (6) I personally know someone who had COVID-19 (yes vs. no); (7) I personally know someone who died of COVID-19; (8) I personally had COVID-19 (yes vs. no); (9) personal existence threatened by COVID-19 pandemic (yes vs. no based on perceived threats to occupational and financial situation); (10) appraisal of negative consequences of SARS-CoV-2 infection [based on the mean score across two items enquiring about the negative consequences of an infection with SARS-CoV-2 with and without regularly wearing a face mask; both rated on a six-point Likert scale ranging from 1 (“very mild”) to 6 (“very serious”)]; (11) fears and worries about the COVID-19 pandemic [mean score across the items “I worry about the coronavirus and the current situation”; “I feel uncomfortable thinking about the coronavirus”; “I fear that I could get severe COVID-19”; “I fear that someone close to me could get severe COVID-19”; “The news and stories I hear about the coronavirus in the media make me nervous or anxious”; all rated on a five-point Likert scale ranging from 1 (“definitely not true”) to 5 (“definitely true”)]; (12) I obtain regular information from health authorities, e.g., Swiss federal office of public health, cantonal health department, WHO (yes vs. no, if information obtained daily or several times per week); (13) I obtain regular information from social media channels, e.g., Facebook, Instagram, Twitter (yes vs. no, if information obtained daily or several times per week); and (14) I trust in medical experts [yes vs. no, if score on a seven-point Likert scale ranging from 1 (“no trust at all”) to 7 (“very high trust”) was at least 5]. A brief description of all predictor variables is given in Table 2.

**Table 2:** Predictor variables associated with non-compliance with non-pharmaceutical interventions to mitigate the spread of SARS-CoV-2 (*n* = 1,157).

		**Crude effect**	**Fully adjusted effect^**#**^**
**Predictor**	**%/mean (SD)**	**B (95%-CI)**	**B (95%-CI)**
Sex	Male (50.4%)	0.09 (0.01 to 0.17)^*^	0.06 (−0.00 to 0.12)
	Female (49.6%)	Reference	Reference
Age (18–90 years)	Mean = 46.3 (SD = 0.70)	−0.01 (−0.01 to −0.01)^***^	−0.01 (−0.01 to −0.00)^***^
Swiss nationality	Yes (85.0%)	−0.04 (−0.15 to 0.08)	0.01 (−0.07 to 0.10)
	No (15.0%)	Reference	Reference
Educational attainment	High (32.0%)	0.08 (−0.01 to 0.18)	0.03 (−0.04 to 0.10)
	Medium (29.0%)	0.02 (−0.08 to 0.12)	−0.02 (−0.09 to 0.05)
	Low (39.1%)	Reference	Reference
Self-perceived high-risk group	Yes (30.3%)	−0.26 (−0.34 to −0.18)^***^	0.03 (−0.05 to 0.11)
	No (69.7%)	Reference	Reference
Personally knows someone who had COVID-19	Yes (50.2%)	−0.04 (−0.12 to 0.04)	−0.04 (−0.10 to 0.03)
	No (49.8%)	Reference	Reference
Personally knows someone who died of COVID-19	Yes (10.2%)	−0.09 (−0.22 to 0.04)	0.03 (−0.06 to 0.12)
	No (89.8%)	Reference	Reference
Personally had COVID-19	Yes (7.4%)	0.15 (−0.01 to 0.32)	0.04 (−0.08 to 0.16)
	No (92.6%)	Reference	Reference
Personal existence threatened by COVID-19 pandemic	Yes (27.8%)	0.05 (−0.04 to 0.14)	0.06 (−0.01 to 0.13)
	No (72.2%)	Reference	Reference
Appraisal of negative consequences of SARS-CoV-2 infection (severity: 1–6)	Mean = 3.51 (SD = 1.28)	−0.17 (−0.20 to −0.15)^***^	−0.07 (−0.10 to −0.04)^***^
Fears and worries about COVID-19 pandemic (severity: 1–5)	Mean = 3.14 (SD = 0.95)	−0.27 (−0.31 to −0.24)^***^	−0.18 (−0.23 to −0.14)^***^
Obtains regular information from health authorities	Yes (35.9%)	−0.30 (−0.37 to −0.22)^***^	−0.14 (−0.20 to −0.08)^***^
	No (64.1%)	Reference	Reference
Obtains regular information from social media channels	Yes (34.0%)	−0.05 (−0.13 to 0.03)	0.02 (−0.05 to 0.08)
	No (66.0%)	Reference	Reference
Trusts in medical experts	Yes (69.2%)	−0.36 (−0.45 to −0.26)^***^	−0.21 (−0.28 to −0.13)^***^
	No (30.8%)	Reference	Reference

# *Includes all predictor variables simultaneously*.

* *p < 0.05*.

^**^p < 0.01.

*** *p < 0.001*.

### Statistical Analysis

To further verify that the individual NPI measures form a unidimensional scale we conducted two principal factor analyses, one with Varimax rotation and another with Promax rotation. We also conducted a series of two-step cluster analyses to examine whether there are distinct groups of people according to non-compliance with specific NPI measures (rather than uniform compliance across individual NPI measures). To do so we conducted a series of models with two to six fixed clusters.

We used generalized linear models with maximum likelihood estimation where non-compliance with NPI was entered as the outcome variable, applying an inverse-Gauss (Wald) distribution and an identity link-function. In a first step we tested all predictor variables separately (univariable model; crude effects) and then, in a second step, entered all variables simultaneously (multivariable model; fully adjusted effects). In a third step, we build a model that included only predictors that were statistically significant in the fully adjusted multivariable model. We did not explore interaction terms due to their many inherent issues arising from power failure, measurement error, multiple testing, and overfitting, ultimately resulting in severely inflated type I errors (15, 16). Non-linear effects (e.g., quadratic, cubic) were tested by categorizing continuous variables through quartile split. The proportion of variance explained was determined with McFadden's pseudo-*R*^2^. The level of statistical significance was set at α = 0.05. We additionally present results based on a Bonferroni correction for multiple testing, where the level of statistical significance was α = 0.004.

All statistical analyses were conducted with SPSS version 28 for Windows.

## Results

**Figure 1:**
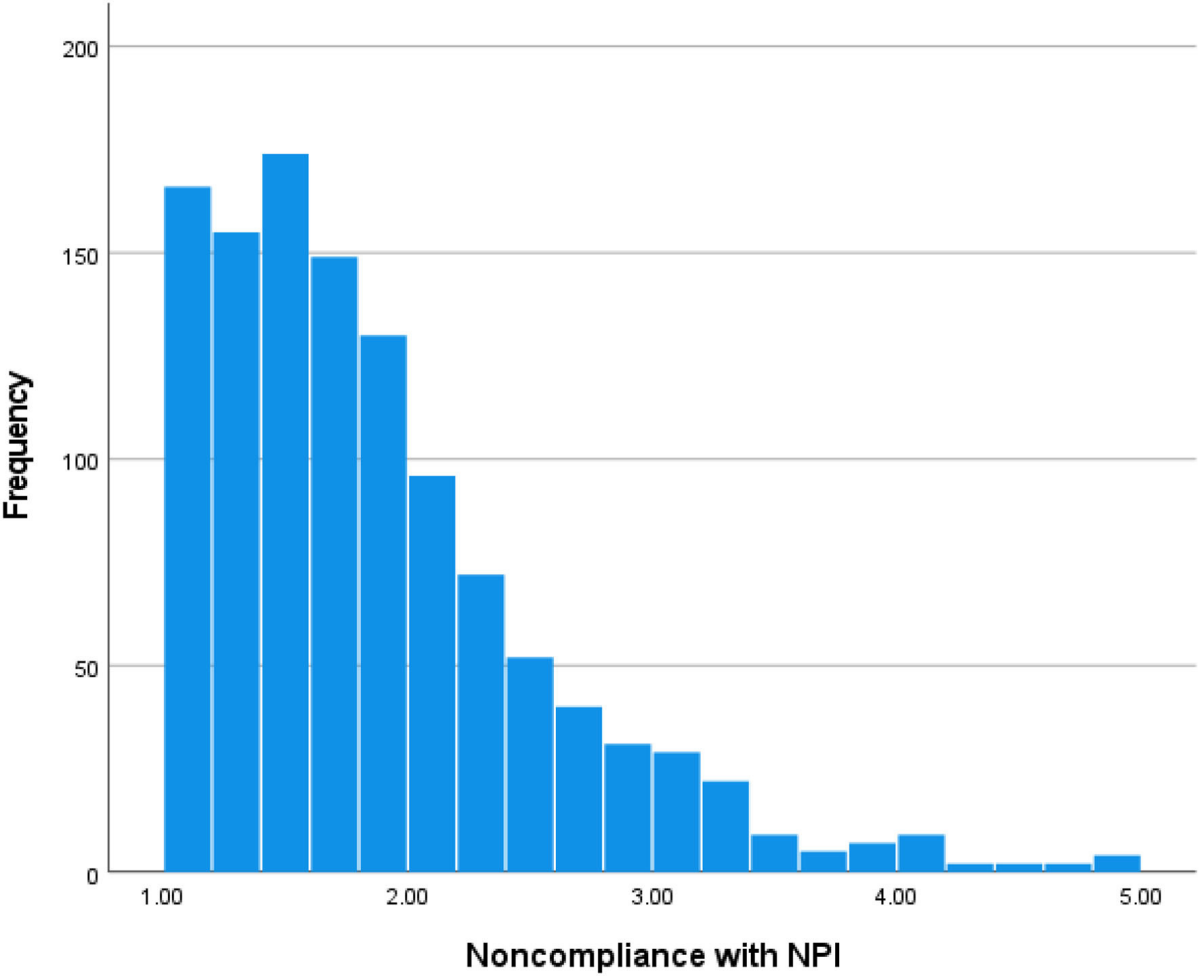
Scores on the global scale of non-compliance with non-pharmaceutical intervention (NPI). Scale ranges from 1 (complete compliance) to 5 (complete non-compliance); scores smaller than 3 indicate a tendency to compliance and scores >3 indicate a tendency to non-compliance.

The sample (*n* = 1,157) consisted of 49.6% women and 50.4% men. The majority (85.0%) was of Swiss nationality, and the mean age was 46.3 years (range: 18–90 years, SD = 16.5 years). More information is provided in Table 2. Non-compliance with individual NPI is shown in Table 1. The highest non-compliance was found with respect to avoiding public transportation (22.7% were rather or definitely noncompliant) and making a SARS-CoV-2 test when having (cold) symptoms (29.9% were rather or definitely noncompliant). Scores on the global measure of non-compliance with NPI ranged from 1 (complete compliance) to 5 (complete non-compliance), with a median score of 1.6 and a modal score of 1. The lower quartile score was 1.3 and the upper quartile score was 2.1. This indicates that about 75% of Swiss people reported good or very good compliance with NPI, but a small minority of about 5% was remarkably noncompliant. A graphical depiction is provided in Figure 1.

Both principal factor analyses and two-step cluster analyses confirmed that the global scale of non-compliance with NPI is unidimensional. The principal factor analyses yielded one latent factor with an eigenvalue >1 onto which all individual NPI measures loaded. The different cluster solutions of the two-step cluster analyses likewise showed that increasing the number of clusters merely captured the uniform degree of non-compliance across all individual NPI measures (e.g., uniformly low, moderate, or high non-compliance). This indicates that people differ based on their uniform level of compliance across all NPI measures. That is, people who are rather noncompliant with a specific public health measure compared to the average person also tend to be relatively noncompliant with any other public health measure.

Out of 14 predictor variables tested, seven were statistically significantly associated with increased non-compliance with NPI (see Table 2). Men were slightly more noncompliant than women. A relatively strong effect was found for age: non-compliance declined with age, indicating that the youngest adults were the most noncompliant. People who self-identified as high-risk group were less noncompliant. People who judged to consequences of an infection with SARS-CoV-2 to be serious and people who were anxious about the pandemic reported considerably lower non-compliance with NPI. Finally, people who obtained regular information from health authorities and people who trusted in medical experts also reported lower non-compliance. Except for sex, all predictor variables remained statistically significant after controlling for multiple testing.

The multivariable model based on all 14 predictor variables accounted for 27.9% of variance explained in non-compliance with NPI. Five predictor variables remained statistically significant at *p* < 0.05, that is, younger age, low appraisal of negative consequences, less fear and worries, not obtaining regular information from health authorities, and not trusting in medical experts. These variables were also significantly related to non-compliance with NPI after correcting for multiple testing (*p* < 0.004). Notably, belonging to a self-perceived high-risk group completely lost its association with non-compliance with NPI after controlling for age and the other predictor variables. We did not detect quadratic or cubic effects. All continuous predictor variables showed linear associations with non-compliance with NPI.

We then build a model that included only the five significant predictor variables from the fully adjusted multivariable model reported above. All predictor variables remained statistically strongly associated with non-compliance with NPI: younger age (*p* < 0.001), low appraisal of negative consequences (*p* < 0.001), less fear and worries (*p* < 0.001), not obtaining regular information from health authorities (*p* < 0.001), and not trusting in medical experts (*p* < 0.001). The regression coefficients with their 95% confidence intervals are shown in Table 3. This more parsimonious five-variable model accounted for 27.2% of variance explained in non-compliance with NPI and the regression coefficients were virtually identical compared to the less parsimonious 14-variable model.

**Table 3:** Final multivariable prediction model of non-compliance with non-pharmaceutical interventions to mitigate the spread of SARS-CoV-2 (*n* = 1,157).

**Predictor**	**B (95%-CI)**	***P***
Age (18–90 years)	−0.006 (−0.008 to −0.005)	<0.001
Appraisal of negative consequences of SARS-CoV-2 infection (severity: 1–6)	−0.070 (−0.098 to −0.041)	<0.001
Fears and worries about COVID-19 pandemic (severity: 1–5)	−0.184 (−0.225 to −0.142)	<0.001
Obtains regular information from health authorities (yes vs. no)	−0.135 (−0.197 to −0.073)	<0.001
Trusts in medical experts (yes vs. no)	−0.203 (−0.279 to −0.127)	<0.001

*McFadden pseudo-R^2^: 0.272*.

## Discussion

Our survey in a representative sample of the Swiss adult general population showed that, after multivariable adjustment, younger age, low appraisal of negative consequences of SARS-CoV-2 infection, low fears and worries about the pandemic, not obtaining regular information from health authorities, and low trust in medical experts, independently predicted non-compliance with NPI to mitigate the spread of SARS-CoV-2. These factors largely replicate the findings from previous studies (10–13). However, in contrast to previous studies, we did not find that men and people with lower educational attainment were more noncompliant than women and people with higher educational attainment (e.g., ref. 10). This could be due to cultural characteristics of the Swiss general adult population, differences in the educational and occupational system, or uncontrolled confounders in previous studies (e.g., fears and worries about COVID-19).

Assuming the detected effects are additive, it follows that young adults who perceive COVID-19 as mostly harmless/inconsequential and who ignore and/or mistrust information from health authorities and medical experts, are the population most likely to be noncompliant with NPI. Given that the vaccines currently available in Switzerland only partially protect against infection with the predominant Delta variant, and that vaccine-induced immunity seems to wane over time (6, 8, 9), these findings have important implications for national efforts to contain SARS-CoV-2 infections and to mitigate the ensuing public health consequences (e.g., overcrowding of intensive care units).

Research has shown that this population of seemingly mistrustful and unconcerned young adults is also hesitant to get a COVID-19 vaccine (17, 18). This group therefore constitutes a high-risk population that is opposed to both vaccines and NPI. Governments and health authorities are advised to concentrate their public campaigns, including both education and intervention programs, on this group. Failure to reach these people may compromise the control (and containment) of the COVID-19 pandemic.

The strength of our study is its large and broadly representative sample and a comprehensive range of objective and/or easily measurable characteristics. However, three limitations need to be taken into account. First and foremost, compliance with NPI fully relied on self-report. Due to social desirability, it is possible that the indicated compliance with NPI deviates from the actual behavior in some people. Second, the survey took place before vaccines were available in Switzerland. Therefore, controlling for current vaccine status may influence the factors associated with non-compliance to NPI. The factors associated with vaccine hesitancy/refusal and non-compliance to NPI are largely similar, but we cannot firmly exclude that controlling for current vaccine status would alter our prediction model. Only a future study with full assessment of vaccination status will be able to answer this crucial question. Third, only adults were included in this study, thus we cannot generalize our findings to children and adolescents. In future studies it would be worthwhile to also assess non-compliance with NPI in minors.

In conclusion, the results of the current study indicate that young adults who are not troubled or anxious about COVID-19, and people who do not obtain information from health authorities and who mistrust medical experts, are the most noncompliant with NPI. These findings may help to target a group of people at high risk of infection and to efficiently concentrate educational and interventional public health efforts to contain the spread of SARS-CoV-2. Future studies that also consider the current vaccination status should preferably assess the reasons for non-compliance with NPI, so that health authorities not only have information in which groups they should intervene, but also how.

## Data Availability Statement

The raw data supporting the conclusions of this article will be made available by the authors, without undue reservation.

## Ethics Statement

Ethical review and approval was not required for the study on human participants in accordance with the local legislation and institutional requirements. The patients/participants provided their written informed consent to participate in this study.

## Author Contributions

MH designed the study, conducted all statistical analyses, and wrote the first manuscript draft. GW and AvW participated in development of the survey questionnaire, data interpretation, and critical revision of the manuscript. All authors contributed to the article and approved the submitted version.

## Conflict of Interest

The authors declare that the research was conducted in the absence of any commercial or financial relationships that could be construed as a potential conflict of interest.

## Publisher's Note

All claims expressed in this article are solely those of the authors and do not necessarily represent those of their affiliated organizations, or those of the publisher, the editors and the reviewers. Any product that may be evaluated in this article, or claim that may be made by its manufacturer, is not guaranteed or endorsed by the publisher.
